# ART in Europe, 2017: results generated from European registries by ESHRE^†^

**DOI:** 10.1093/hropen/hoab026

**Published:** 2021-08-05

**Authors:** The European IVF-Monitoring Consortium (EIM) for the European Society of Human Reproduction and Embryology (ESHRE), Orion Gliozheni, Eduard Hambartsoumian, Heinz Strohmer, Obruca & Strohmer Partnerschaft Goldenes Kreuz-Kinderwunschzentrum, Elena Petrovskaya, Oleg Tishkevich, Kris Bogaerts, Christine Wyns I-Biostat, Devleta Balic, Sanja Sibincic, Irena Antonova, Hrvoje Vrcic, Dejan Ljiljak, Karel Rezabek, Jitka Markova, Josephine Lemmen, Deniss Sõritsa, Mika Gissler, Sari Pelkonen, Bilal Majed, Jacques de Mouzon, Andreas Tandler, Nikos Vrachnis, Janos Urbancsek, G Kosztolanyi, Hilmar Bjorgvinsson, Giulia Scaravelli, Roberto de Luca, Vyacheslav Lokshin, Sholpan Karibayeva, Valeria Magomedova, Raminta Bausyte, Ieva Masliukaite, Caroline Schilling, Jean Calleja-Agius, Veaceslav Moshin, Tatjana Motrenko Simic, Dragana Vukicevic, Jesper M J, Zoranco Petanovski, Liv Bente Romundstad, Anna Janicka, Carlos Calhaz, Joana Maria Mesquita Guimaraes, Ana Rita Laranjeira, Ioana Rugescu, Bogdan Doroftei, Vladislav Korsak, Snezana Vidakovic, Irma Virant-Klun, Irene Cuevas Saiz, Fernando Prados Mondéjar, Christina Bergh, Maya Weder, Marco Buttarelli, Marie-Pierre Primi, Basak Balaban, Timur Gürgan, Richard Baranowski, Mykola Gryshchenko, C Wyns, Ch De Geyter, C Calhaz-Jorge, M S Kupka, T Motrenko, J Smeenk, C Bergh, A Tandler-Schneider, I A Rugescu, S Vidakovic, V Goossens

**Affiliations:** 1 Cliniques Universitaires Saint-Luc, Université Catholique de Louvain, Brussels, Belgium; 2 Reproductive Medicine and Gynecological Endocrinology (RME), University Hospital, University of Basel, Basel, Switzerland; 3 Faculdade de Medicina da Universidade de Lisboa, Lisboa, Portugal; 4 Fertility Center—Gynaekologicum, Hamburg, Germany; 5 Human Reproduction Center Budva, Budva, Montenegro; 6 Elisabeth Twee Steden Ziekenhuis, Tilburg, The Netherlands; 7 Department of Obstetrics and Gynecology, Institute of Clinical Sciences, Göteborg University, Göteborg, Sweden; 8 Fertility Center Berlin, Berlin, Germany; 9 National Transplant Agency, Romania; 10 Clinical Center Serbia «GAK», Institute of Obstetrics and Gynecology, Beograd, Serbia; 11 ESHRE Central Office, Grimbergen, Belgium

**Keywords:** IVF, ICSI, IUI/, egg donation, frozen embryo replacement, surveillance, vigilance, registry, data collection/ fertility preservation

## Abstract

**STUDY QUESTION:**

What are the data on ART and IUI cycles, and fertility preservation (FP) interventions reported in 2017 as compared to previous years, as well as the main trends over the years?

**SUMMARY ANSWER:**

The 21st ESHRE report on ART and IUI shows the continual increase in reported treatment cycle numbers in Europe, with a decrease in the proportion of transfers with more than one embryo causing an additional slight reduction of multiple delivery rates (DR) as well as higher pregnancy rates (PR) and DR after frozen embryo replacement (FER) compared to fresh IVF and ICSI cycles, while the number of IUI cycles increased and their outcomes remained stable.

**WHAT IS KNOWN ALREADY:**

Since 1997, ART aggregated data generated by national registries, clinics or professional societies have been gathered and analyzed by the European IVF-monitoring Consortium (EIM) and communicated in a total of 20 manuscripts published in *Human Reproduction* and *Human Reproduction Open*.

**STUDY DESIGN, SIZE, DURATION:**

Data on European medically assisted reproduction (MAR) are collected by EIM for ESHRE on a yearly basis. The data on treatments performed between 1 January and 31 December 2017 in 39 European countries were provided by either National Registries or registries based on personal initiatives of medical associations and scientific organizations.

**PARTICIPANTS/MATERIALS, SETTING, METHODS:**

Overall, 1382 clinics offering ART services in 39 countries reported a total of 940 503 treatment cycles, including 165 379 with IVF, 391 379 with ICSI, 271 476 with FER, 37 303 with preimplantation genetic testing (PGT), 69 378 with egg donation (ED), 378 with IVM of oocytes, and 5210 cycles with frozen oocyte replacement (FOR). A total of 1273 institutions reported data on 207 196 IUI cycles using either husband/partner’s semen (IUI-H; n = 155 794) or donor semen (IUI-D; n = 51 402) in 30 countries and 25 countries, respectively. Thirteen countries reported 18 888 interventions for FP, including oocyte, ovarian tissue, semen and testicular tissue banking in pre- and postpubertal patients.

**MAIN RESULTS AND THE ROLE OF CHANCE:**

In 21 countries (20 in 2016) in which all ART clinics reported to the registry, 473 733 treatment cycles were registered for a total population of approximately 330 million inhabitants, allowing a best-estimate of a mean of 1435 cycles performed per million inhabitants (range: 723–3286).

Amongst the 39 reporting countries, the clinical PR per aspiration and per transfer in 2017 were similar to those observed in 2016 (26.8% and 34.6% vs 28.0% and 34.8%, respectively). After ICSI the corresponding rates were also similar to those achieved in 2016 (24% and 33.5% vs 25% and 33.2% in 2016). When freeze all cycles were removed, the clinical PRs per aspiration were 30.8% and 27.5% for IVF and ICSI, respectively.

After FER with embryos originating from own eggs the PR per thawing was 30.2%, which is comparable to 30.9% in 2016, and with embryos originating from donated eggs it was 41.1% (41% in 2016). After ED the PR per fresh embryo transfer was 49.2% (49.4% in 2016) and per FOR 43.3% (43.6% in 2016).

In IVF and ICSI together, the trend towards the transfer of fewer embryos continues with the transfer of 1, 2, 3 and ≥4 embryos in 46.0%, 49.2%, 4.5% and in 0.3% of all treatments, respectively (corresponding to 41.5%, 51.9%. 6.2% and 0.4% in 2016). This resulted in a reduced proportion of twin DRs of 14.2% (14.9% in 2016) and stable triplet DR of 0.3%. Treatments with FER in 2017 resulted in a twin and triplet DR of 11.2% and 0.2%, respectively (vs 11.9% and 0.2% in 2016).

After IUI, the DRs remained similar at 8.7% after IUI-H (8.9% in 2016) and at 12.4% after IUI-D (12.4.0% in 2016). Twin and triplet DRs after IUI-H were 8.1% and 0.3%, respectively (in 2016: 8.8% and 0.3%) and 6.9% and 0.2% after IUI-D (in 2016: 7.7% and 0.4%). Amongst 18 888 FP interventions in 13 countries, cryopreservation of ejaculated sperm (n = 11 112 vs 7877 from 11 countries in 2016) and of oocytes (n = 6588 vs 4907 from eight countries in 2016) were the most frequently reported.

**LIMITATIONS, REASONS FOR CAUTION:**

As the methods of data collection and levels of reporting vary amongst European countries, interpretation of results should remain cautious. Some countries were unable to deliver data about the number of initiated cycles and deliveries.

**WIDER IMPLICATIONS OF THE FINDINGS:**

The 21st ESHRE report on ART, IUI and FP interventions shows a continuous increase of reported treatment numbers and MAR-derived livebirths in Europe. Being already the largest data collection on MAR in Europe, efforts should continue to optimize data collection and reporting with the perspective of improved quality control, transparency and vigilance in the field of reproductive medicine.

**STUDY FUNDING/COMPETING INTEREST(S):**

The study has received no external funding and all costs are covered by ESHRE. There are no competing interests.

## Introduction

This is the 21st annual report of the European IVF-monitoring Consortium (EIM) under the umbrella of ESHRE, assembling the data on ART, IUI and fertility preservation (FP) reported by 39 participating European countries in 2017 (Supplementary Table SI).

Eighteen previous annual reports published in *Human Reproduction* (https://www.eshre.eu/Data-collection-and-research/Consortia/EIM/Publications.aspx) and two in *Human Reproduction Open* (De Geyter *et al.*, 2020; Wyns *et al.*, 2020) covered treatment cycles from 1997 to 2016. As in previous reports, the printed version contains the five most relevant tables. Twenty additional supplementary tables (Supplementary Tables SI–SXX) are available online on the publisher's homepage. The presentation of the data is consistent with those published in previous reports to allow easy comparison and assessment of trends. For the second consecutive year, data on FP were collected and added to this report.

## Material and methods

Data collected on an aggregate basis were provided by 39 European countries, covering treatments with IVF, ICSI, frozen embryo replacement (FER), egg donation (ED), IVM, preimplantation genetic testing (PGT; pooled data) and frozen oocyte replacement (FOR). With regards to IUI, data for use of husband’s/partner’s semen (IUI-H) and donor semen (IUI-D) were distinguished. The report includes treatments started between 1 January and 31 December in 2017. Data on pregnancies and deliveries represent the outcomes of treatments performed in 2017. Data on FP, including numbers and types of cryopreserved material and interventions for use of cryostored material between 1 January and 31 December in 2017, were provided and reported as aggregated data of events that occurred during a 1-year period.

The national representatives of the 44 countries that are members of the EIM were asked to fill out questionnaires. The same data sets as in 2016 for a total of 10 specific modules were sent using software designed for the requirements of this data collection (Dynamic Solutions, Barcelona, Spain). Any detected inconsistencies were clarified through contacts between the administrator of the ESHRE central office, V.G., and the national representative.

The data were analyzed and presented similarly to previous reports, with some additional subgroups of interventions since the report on 2016 data, and footnotes to the tables were added for clarification on diverging results reported by individual countries, when applicable.

The terminology used was based on the glossary of The International Committee for Monitoring Assisted Reproductive Technology (Zegers-Hochschild *et al*., 2017).

## Results

### Participation and data completeness

Table I shows the number of clinics offering ART services with all available treatment modalities and institutions performing IUI (IUI-H and IUI-D). Compared to 2016, the total number of reporting clinics (1381 in 2017 vs 1347 in 2016) and number of reported treatments (940 503 in 2017 vs 918 159 in 2016, +2.4%) increased. Amongst the 51 European countries, 44 are EIM members including 28 that are members of the European Union and 39 (40 in 2016) provided data (Supplementary Table SI). Non-EIM members are mainly small countries not offering ART services. Cyprus, Georgia, Ireland, Slovakia and Turkey failed to deliver data (11.3% of EIM members, as in 2016). In 21 countries (53.8% of reporting countries, 45% in 2016) all of the ART clinics within the country reported data sets. Amongst 1531 known IVF clinics in Europe, 1381 clinics reported their data (90.1%; 91.8% in 2016). Similar to 2016, the four European countries with the largest treatment numbers in 2017 were Spain (125 592; 140 909 in 2016), Russia (137 211; 121 232 in 2016), France (108 820; 104 773 in 2016) and Germany (99 466; 99 226 in 2016).

**Table I:** Treatment frequencies after ART in European countries in 2017.

	IVF clinics in the country												Cycles/million*	
Country	IVF Clinics	Included IVF clinics	IUI labs	Included IUI labs	IVF	ICSI	FER	PGD	ED	IVM	FOR	All	Women 15-45	Population
Albania	11	1	11	1	0	105	82	1	24	0	2	214		
Armemia	6	5	9	5	513	642	675	0	459	0	15	2304		
Austria	29	29	0	0	1702	5298	2801	0	218	0	0	10 019	5870	1137
Belarus	8	7	10	7	1171	1570	663	68	61	0	9	3542		
Belgium	18	18	28	28	2819	13 133	12 881	1145	1334	149	76	31 537	14 411	2778
Bosnia-Herzegovina	6	2	6	2		82	80	0	0	0	0	162		
Bulgaria	36	36	37	37	311	4644	1365	124	714	0	0	7158	4719	1015
Croatia	15	15	16	16	1607	2727	1389	1436	0	0	62	7221	8864	1682
Czech Republic	44	44	0	0		15 557	13 907	0	5328	0	0	34 792	16 547	3286
Denmark	23	23	51	50	7262	6394	2726	345	798	0	19	17 544	15 783	3045
Estonia	6	6	6	6	637	1203	890	13	216	0	0	2959	11 705	2249
Finland	16	16	21	21	2471	1790	3584	97	645	0	0	8587	7519	1558
France	103	103	183	183	23 538	44 165	37 469	1452	1459	120	617	108 820	8528	1620
Germany	134	127	0	0	18 679	53 290	27 234	0	0	0	263	99 466		
Greece	37	29	37	29	2166	13 588	5192	1534	5008	9	50	27 547		
Hungary	14	13	0	0	1386	4022	0	0	23	0	0	5431		
Iceland	1	1	1	1	259	221	267	0	65	0	0	812	11 498	2400
Italy	204	204	366	366	8049	44 965	17 281	3133	4864	0	1391	79 683	7335	1316
Kazakhstan	15	8	0	0	1664	3497	2416	1422	1103	0	0	10 102		
Latvia	6	3	6	3	265	581	549	2	159	0	15	1571		
Lithuania	6	5	6	5		395	187	2	0	0	0	584		
Luxembourg	1	1	0	0	260	457	429	0	0	0	0	1146	9482	1904
Malta	2	2	3	0	17	214	0	0	0	0	155	386	3893	811
Moldova	4	3	5	0		960	346	0	0	0	0	1306		
Montenegro	5	4	5	4	0	582	84	0	0	0	0	666		
North-Macedonia	7	5	2	0	421	2100	277	0	142	0	0	2940		
Norway	11	11	10	10	3963	2997	4476	0	0	0	0	11 436	11 566	2175
Poland	42	42	0	39	465	14 100	10 390	1242	1257	18	335	27 807	4712	723
Portugal	24	24	26	26	2646	3681	2317	275	1292	5	48	10 264	5387	997
Romania	19	10	19	10	1168	1732	1452	14	29	0	2	4397		
Russia	220	159	0	0	38 874	48 739	35 979	5228	7777	20	594	137 211		
Serbia	18	1	18	1	147	75	34	0	0	0	1	257		
Slovenia	3	3	2	2	1054	2092	1422	62	5	0	4	4639	8986	2245
Spain	247	239	366	301	6473	43 790	27 690	15 373	31 441	27	798	125 592		
Sweden	17	17	0	0	6187	5906	7006	504	267	0	0	19 870		
Switzerland	27	27	0	0	986	5013	4944	76	0	0	0	11 019	6595	1299
The Netherlands	13	13	0	0	6417	7574	13 469	497	0	0	0	27 957	8936	1637
Ukraine	48	40	17	17	849	10 904	9080	1634	1134	0	7	23 608		
UK	85	85	103	103	20 953	22 594	20 443	1624	3556	30	747	69 947	5392	1080
**All**	1531	1381	1370	1273	165 379	391 379	271 476	37 303	69 378	378	5210	940 503	7662	1435

Treatment cycles in IVF and ICSI refer to initiated cycles.

For Austria, Belgium and Iceland treatment cycles refer to aspirations. For Austria, Belgium and Lithuania the total number of initiated cycles was only available for IVF and ICSI together, being 10 216, 18 681 and 395, respectively.

For the Czech Republic and Lithuania, no distinction between IVF and ICSI is made. All cycles are counted as ICSI.

Treatment cycles in frozen embryo replacement (FER) refer to thawings.

For Croatia, Finland, Sweden and the Netherlands, treatment cycles refer to transfers.

Treatment cycles in PGD contain both fresh and frozen cycles and refer to initiated cycles in the fresh cycles and aspirations in the frozen cycles.

Treatment cycles in egg donation (ED) refer to donation cycles and contain fresh and frozen cycles.

ED fresh: For Bulgaria, France and Iceland treatment cycles refer to aspirations.

Treatment cycles in IVM refer to aspirations.

Treatment cycles in frozen oocyte replacement (FOR) refer to thawings, for Finland it refers to transfers.

### Size of the clinics and reporting methods

The size of reporting clinics between and inside countries, defined by the number of treatment cycles, remains highly variable (Supplementary Table SII). In 2017, clinics with cycle numbers between 200–499 and 500–999 were the most common (25.9% and 26.3%, respectively vs 29.5% and 26%). The proportion of clinics performing more than 1000 treatment cycles per year is comparable to 2016 (18.9% vs 19.4% in 2016). Small clinics providing less than 100 treatments cycles per year were present in 24 countries (57% of the countries).

Registry requirements and reporting methods for each country are presented in Supplementary Table SIII. Data collection was either voluntary (15 out of 39 countries) or compulsory. Nineteen countries had only a partial reporting and provided the data mainly on a voluntary basis (15/19 countries) to medical organizations (9 countries), to the national health authority (2 countries) or as a single person who took the initiative (3 countries). One country reported as personal initiative and to the National Health Authority and one country to a medical organization and the National Health Authority.

By contrast, complete reporting was mostly achieved when data collection was compulsory (20/21 countries) with subsequent data communication to the national health authority (all but four countries). Transfer of data was mostly done on an aggregate basis (24 countries/39).

### Number of treatment cycles per technique and availability

**Figure 1.**
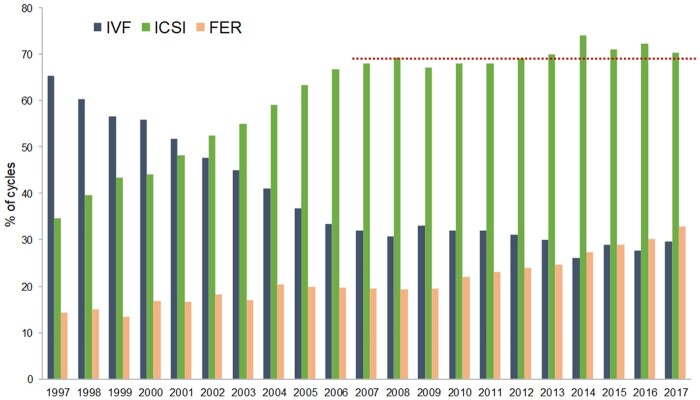
**Proportion of IVF versus ICSI and frozen embryo replacement (FER) in Europe, 1997–2017**.

In 2017, 940 503 treatment cycles were reported to EIM (22 344 more than in 2016, +2.4%). Since 1997, increasing numbers of clinics reported to EIM to reach a total 10 713 407 treatments cycles and the birth of more than 2 059 975 infants (Table II). As seen in Table I, 10 countries reported fewer treatment cycles and, compared to 2016, Croatia was now able to provide data, whereas Ireland and Cyprus not. Furthermore, the largest increments in reported treatment numbers were observed for Kazakhstan (+ 5642, +3 clinics) and Russia (+15 976, +8 clinics). Table I shows the numbers of treatment cycles per technique in 2017: ICSI remains the most used (391 379, 41.6% of all treatment cycles versus 407 222, 44.4% in 2016). Cycles of IVF, FER, ED, FOR, PGT and IVM represented 17.6%, 28.9%, 7.4%, 0.5%, 4% and 0.0004% of all cycles, respectively. While the distribution of the available techniques remained similar to 2016 (respectively, 17%, 27%, 8.1%, 0.5%, 2.9% and 0.0007%), reported cycle numbers increased for IVF, FER, FOR and PGT and decreased for ICSI, ED and IVM. The steepest rise in treatment numbers was observed for PGT (+37.8%, + 27.4% in 2016) and FER (+8.2%, +13.9% in 2016). However, while in 2016, ED and IVM cycle numbers increased (+14.7% and +246.8%, respectively), a decrease of 6.1% and 42.2% was observed in 2017. The proportion of FER relative to fresh treatments (IVF+ICSI) is still on the rise (48.9%, versus 44.1% in 2016, 40.3% in 2015 and 37.8% in 2014). Denmark, Hungary and Malta did not report FER in 2017. The highest proportions of FER treatments (calculated as FER/(FER+ICSI+IVF)) were reached in Bosnia-Herzegovina (49.4%), The Netherlands (49%), Czech Republic (47.2%), Finland (45.7%), Switzerland (45.2%), Belgium (44.7%), Albania (43.9%) and Ukraine (43.6%), with an overall proportion of 32.6% (Fig. 1).

**Table II:** Number of institutions offering ART services, treatment cycles and infants born after ART in Europe, 1997–2017.

Year	Countries	Clinics	Cycles	Cycle increase (%)	Infants born
1997	18	482	203 225		35 314
1998	18	521	232 225	+14.3	21 433
1999	21	537	249 624	+7.5	26 212
2000	22	569	275 187	+10.2	17 887
2001	23	579	289 690	+5.3	24 963
2002	25	631	324 238	+11.9	24 283
2003	28	725	365 103	+12.6	68 931
2004	29	785	367 056	+0.5	67 973
2005	30	923	419 037	+14.2	72 184
2006	32	998	458 759	+9.5	87 705
2007	33	1029	493 420	+7.7	96 690
2008	36	1051	532 260	+7.9	107 383
2009	34	1005	537 463	+1.0	109 239
2010	31	991	550 296	+2.4	120 676
2011	33	1314	609 973	+11.3	134 106
2012	34	1354	640 144	+4.9	143 844
2013	38	1169	686 271	+7.2	149 466
2014	39	1279	776 556	+13.1	170 163
2015	38	1343	849 811	+10.2	187 542
2016	40	1347	918 159	+8.0	195 766
2017	39	1382	940 503	+2.4	198 215

Total			10 713 407		2 059 975

Figure 1 shows the evolution and continuing preponderance of ICSI over conventional IVF. Amongst a total of 556 758 fresh treatments (ICSI+IVF), 70.3% (72.3% in 2016) were performed with ICSI.

The number of cycles per million women of reproductive age and per million inhabitants is shown in Table I and Supplementary Table SIV. Availability of ART was calculated for the 21 countries with full coverage (Supplementary Table SIV). While there is a huge variability in availability when all techniques are considered (range: 3893–16 547 per million women aged 15–45 years), ART was most available in Czech Republic and least available in Malta. Hence, corresponding proportions of newborns resulting from ART were 5.4% and 1.1% of all newborns in these countries. Other countries that reported high proportions were Denmark (5.6%), Iceland (5.6%), Estonia (5.3%) and Slovenia (5.3%).

### Pregnancies and deliveries after treatment

Table III shows pregnancy and delivery rates (DR) after IVF or ICSI and after FER (after both IVF and ICSI). As numbers of initiated cycles have constantly been incompletely reported, outcome data were calculated per aspiration.

**Table III:** Results after ART in 2017.

	IVF				ICSI			FER				
Country	Initiated Cycles IVF + ICSI	Aspirations	Pregnancies per aspiration (%)	Deliveries per aspiration (%)	Aspirations	Pregnancies per aspiration (%)	Deliveries per aspiration (%)	Thawings FER	Pregnancies per thawing (%)	Deliveries per thawing (%)	ART infants	ART infants per national births (%)
Albania	105	0			105	37.1	29.5	82	28.0	20.7	65	
Armenia	1155	492	42.3	40.9	636	38.2	36.8	675	36.7	33.0	884	2.2
Austria	10 216	1702	31.6	28.0	5298	28.5	24.4	2801	32.9	28.4	2824	3.2
Belarus	2741	1117	36.5	27.6	1511	35.3	25.8	663	39.1	24.7	1066	1.0
Belgium	18 681	2819	24.1	18.0	13 133	22.2	15.8	12 881	27.1	18.8	5711	4.8
Bosnia-Herzegovina, Federation part	82				72	25.0	23.6	80	38.7	20.0	172	
Bulgaria	4955	311	30.9	21.9	4644	20.3	14.9	1365	28.4	21.1		
Croatia	4334											
Czech Republic	15 557				15 122	22.4	14.0	13 907	29.7	17.7	6191	5.4
Denmark	13 656	6525	22.6	13.1	5659	23.5	16.9				3411	5.6
Estonia	1840	620	28.1	21.6	1199	29.6	21.9	890	27.9	18.8	725	5.3
Finland	4261	2341	22.9	17.5	1679	21.0	16.6					
France	67 703	20 768	21.0	17.7	41 412	21.2	18.3	37 469	22.9	19.2	20 966	2.7
Germany	71 969	17 771	25.1	17.8	50 863	26.6	19.0	27 234	26.5	17.7	21 292	
Greece	15 754	2166	24.9	18.1	13 588	18.4	12.7	5192	43.4	24.2	6276	
Hungary	5408	1326			4003							
Iceland		259	28.2	25.5	221	26.7	21.7	267	41.9	32.2	229	5.6
Italy	53 014	7201	21.8	14.8	40 710	19.0	12.2	17 281	29.3	20.2	12 638	2.8
Kazakhstan	5161	1285	24.3	14.1	3497	23.7	13.1	2416	49.1	26.5	1700	5.7
Latvia	846	265	28.7	20.8	581	25.3	18.2	549	43.0	29.5	401	
Lithuania	395							187	36.4	3.2	125	0.4
Luxembourg	717	234	28.6	20.5	419	27.9	21.2	429	29.1	17.2	243	3.9
Malta	231	17			204						48	1.1
Moldova	960				891	33.8	24.7	346	38.7	21.4		
Montenegro	582				575	24.3	19.0	84	26.2	23.8	170	2.3
North-Macedonia	2521	385	34.0	10.4	1885	32.9	27.5	277	41.9	31.0	747	4.7
Norway	6960	3759	24.6	20.8	2856	23.7	20.5	4476	22.0	17.8		
Poland	14 565	465	27.7	22.2	13 923	26.6	16.3	10 390	35.8	22.0	5680	1.4
Portugal	6327	2521	27.4	20.5	3490	21.7	16.1	2317	33.7	24.8	2436	2.8
Romania	2900	1138	26.9	20.3	1631	25.6	20.4	1452	33.5	26.4	1105	0.6
Russia	87 613	37 275	29.2	21.2	47 500	25.1	17.4	35 979	40.2	27.7	34 173	2.0
Serbia	222	146	31.5	26.0	74	29.7	24.3	34	41.2		74	
Slovenia	3146	1010	30.5	25.0	2047	23.4	18.8	1422	33.1	24.3	1063	5.3
Spain	50 263	5831	25.3	18.8	38 607	21.3	15.6	27 690	34.9	24.6	30 898	7.9
Sweden	12 093	5823	29.3	25.0	5540	26.2	21.7				5284	4.5
Switzerland	5999	907	23.7	18.2	4629	23.9	18.2	4944	25.3	17.8	2195	2.5
The Netherlands	13 991	5536	29.6	21.7	6500	33.5	25.8					
Ukraine	11 753	826	35.4	28.1	10 690	22.6	17.9	9080	48.4	37.8	7728	1.9
UK	43 547	18 409	32.0	28.0	22 402	33.3	29.2	20 443	35.9	31.2	22 047	2.9
**All**	562 223	151 250	26.8	20.5	367 796	24.0	17.7	243 302	32.2	23.1	198 567	3.1

Total rates refer to these countries where all data were reported for the given technique:.

^†^ ART infants also include ED.

For IVF and ICSI there were for Belarus, Czech Republic, Denmark, Finland, France, Greece, Latvia, Lithuania, Poland, Portugal, Russia and Sweden respectively 35, 18,3 688, 16, 8, 10, 9, 220, 6, 1303 and 14 deliveries with unknown outcome. These were accepted as singletons to calculate the ART infants.

For FER there were for Austria, Czech Republic, Finland, France, Greece, Kazakhstan, Latvia, Poland, Portugal, Russia and Sweden respectively 796, 16, 849, 9, 7, 2, 31, 167, 4, 1100 and1 deliveries with unknown outcome. These were accepted as singletons to calculate the ART infants.

For ED there were for Belarus, Czech Republic, Finland, Greece, Kazakhstan, Latvia, Portugal, Russia and Spain respectively 3, 27, 147, 2, 2, 8, 1, 153 and 1221 deliveries with unknown outcome. These were accepted as singletons to calculate the ART infants.

For PGD there were for Finland, France, Portugal and Russia respectively 37, 2, 1 and 26 deliveries with unknown outcome. These were was accepted as singleton to calculate the ART infants.

In the Czech Republic, IVF and ICSI were reported together under ICSI. In Lithuania, IVF and ICSI were reported together, no details on pregnancies and deliveries.

Amongst the 39 reporting countries, 35 were able to provide both pregnancy and delivery data per aspiration after IVF (n = 30) and/or ICSI (n = 35). For FER when considering thawing cycles, 29 and 28 countries were able to report pregnancy and delivery rates, respectively. Supplementary Table SIV shows the numbers of deliveries for the 21 countries with full coverage of the reporting.

Pregnancy and delivery rates (for all types of treatment cycles) varied significantly from one country to another, as in previous years.

Per aspiration, pregnancy rates (PR) ranged from 18.4% to 42.3% and DR from 10.4% to 40.9% in fresh cycles after IVF or ICSI (when considering data from countries able to provide data excluding the freeze-all cycles). Pregnancy and delivery rates per thawing for FER varied between 22% and 49.1% and between 3.2% and 37.8%, respectively. Overall, pregnancy and delivery rates were higher for FER cycles (per thawing) than for both fresh IVF and ICSI cycles (per aspiration) (Table III).

When considering the stage of replaced embryos, data showed PR for blastocyst transfers to be higher (38.5% vs 27.2% for cleavage stage embryos for FER and 41.7% vs 29.4% for cleavage stage embryos, for fresh IVF and ICSI cycles, respectively).

For the fourth time, «freeze all» cycles were collected (Supplementary Tables SV and SVI) including either freezing of all oocytes reported by 10 countries for IVF (10 in 2016 and 6 in 2015) and 17 countries for ICSI (15 in 2016 and 14 in 2015), or of all embryos by 22 countries for IVF (22 in 2016 and 21 in 2015) and 27 countries for ICSI (22 in 2016 and 24 in 2015). The highest proportions of freeze all cycles per aspiration for oocytes and for embryos together were 5.4% (IVF) (5.1% in 2016) and 49.1% (ICSI) (28.9% in 2016), respectively.

Cycle numbers, aspirations, transfers, pregnancies, deliveries in IVF, ICSI and FER (after both IVF and ICSI) by country are presented in the Supplementary Tables SV–SVII.

ED cycle numbers were available for 21 countries (26 in 2016) although 26 (29 in 2016) provided outcome data (Supplementary Table SVIII). Most ED cycles were reported from Spain, the Czech Republic and Russia. The number of aspirations of donated oocytes was 34 443 (33 406 in 2016) that led to 26 447 fresh transfers (28 451 in 2016), while the replacements of frozen oocytes (FOR) were 14 129 (11 757 in 2016). The PR per embryo transfer were 49.2% (49.4% in 2016) for freshly donated oocytes and 41.1% (41% in 2016) for thawed oocytes although a high variability was seen between countries, ranging from 0% (2 cycles) to 61.6% for fresh oocytes and from 23.1% to 62.2% for thawed oocytes. Overall, 21 137 deliveries were reported (22 497 in 2016 and 19 849 in 2015) and pregnancy and delivery rates per transfer were higher than in cycles with own gametes (partner donation) both for fresh (IVF and ICSI) and FER cycles.

### Age distribution

As seen in Supplementary Tables SIX and SX, age distributions of women treated with IVF and ICSI varied between countries. Some countries were not able to provide age categories (10 for IVF and six for ICSI). The highest percentage of women aged 40 years and older undergoing aspiration for IVF was found in Greece (as in 2016), whereas the highest percentage of women aged <34 years was found in Ukraine (Montenegro in 2016). For ICSI the highest percentage of women aged 40 years and older undergoing aspiration was found in Greece (as in 2016), whereas the highest percentage of women undergoing aspiration aged <34 years was recorded in Albania (in Kazakhstan in 2016 and Albania in 2015). An age-dependent decrease of pregnancy and delivery rates for IVF and ICSI cycles was reported as expected, with pregnancy and delivery rates in women aged 40 years and older ranging between 5.9% and 32.5%, and 0% and 20%, respectively. Concerning FER (Supplementary Table SXI), the age related decline was still visible and DR recorded amongst women aged 40 years and older were between 0 and 36.4%.

In ED cycles (Supplementary Table SXII), the age of the recipient women had no influence on outcomes.

### Numbers of embryos transferred and multiple births

**Figure 2.**
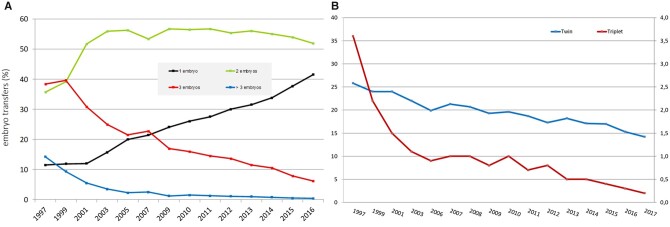
**Embryo transfer and multiple births in Europe, 1997–2017. (A)** Number of embryos transferred in IVF and ICSI during fresh cycles**. (B)** Percentages of twin and triplet deliveries.

Subgroups defined by the number of embryos replaced per transfer procedure after IVF and ICSI together as well as multiple birth rates are presented in Table IV. Six countries did not report on the number of replaced embryos or on multiplicity. While overall most transfers involved the replacement of two embryos (49.2%, 51.9% of the transfer cycles in 2016), the proportion of transfers of only one embryo per cycle is still on the rise (46% vs 41.5% in 2016), and the number of transfers of three or more embryos continued to decrease (Fig. 2A). Thirteen countries reported more than 50% of single embryo transfers (elective or not) (10 in 2016) (same 10 as in 2016 plus Estonia, France and Latvia). For the first time, none of the reporting countries carried out more than 50% of their transfers with three embryos (in 2016 only Serbia did). The highest proportion of transfers of four or more embryos was recorded in Greece (4% vs 4.2% in 2016). For the second consecutive year, the embryo stage at transfer was collected. Taking into account that the embryo stage at transfer was unknown for 23.2% of the fresh (IVF+ICSI) cycles, 44.1% (41.9% in 2016) of the transfers were performed at the blastocyst stage. The corresponding figure for FER was 64.1% (62.2% in 2016). Such information was not available for each of the subgroups for numbers of embryos replaced.

**Table IV:** Number of embryos transferred after ART and deliveries in 2017.

	IVF + ICSI														FER	
Country	Transfers	Fresh Transfers cleavage stage	Fresh Transfers blastocyst stage	Fresh Transfers Unkown stage	% Fresh transfers cleavage stage**	% Fresh transfers blastocyst stage**	1 embryo (%)	2 embryos (%)	3 embryos (%)	4+ embryos (%)	Deliveries	Twin (%)	Triplet (%)	Deliveries	Twin (%)	Triplet (%)
Albania	90	90	0	0	100.0	0.0	12.2	85.6	2.2	0.0	31	25.8	0.0	17	0.0	0.0
Armemia	1109	501	608	0	45.2	54.8	32.9	61.1	6.0	0.0	435	9.7	0.0	223	0.9	0.0
Austria	8646	2499	6147	0	28.9	71.1	69.6	30.2	0.2	0.0	2565	9.6	0.2	796		
Belarus	2325	1374	951	0	59.1	40.9	20.7	71.1	8.2	0.0	698	21.1	0.8	164	9.8	0.6
Belgium	12 328	8389	3939	0	68.0	32.0	67.9	28.1	3.4	0.5	2587	7.5	0.0	2422	6.4	0.1
Bosnia-Herzegovina	494	393	101	0	79.6	20.4	15.4	43.7	39.9	1.0	115	27.0	2.6	16	25.0	0.0
Bulgaria	2986			2986												
Croatia	3930			3930			45.8	49.6	4.6	0.0						
Czech Republic	10 887			10 887			73.7	25.9	0.5	0.0	2119	6.3	0.1	2460	6.8	0.2
Denmark	9253	6648	2596	9	71.9	28.1	75.8	24.0	0.2	0.0	1812	4.3	0.0	1307	2.4	0.0
Estonia	1493	855	638	0			51.8	43.6	4.6	0.0	397	12.6	0.3	167	15.6	0.0
Finland	2881			2881			91.3	8.7	0.0	0.0	688			849		
France	44 318	30 250	14 068	0	68.3	31.7	50.8	45.2	3.7	0.3	11 268	11.6	0.2	7189	7.2	0.0
Germany	54 255	34 789	19 466	0	64.1	35.9	25.1	69.5	5.4	0.0	12 813	21.3	0.7	4808	15.0	0.4
Greece	7985	4971	3014	0			21.3	61.8	12.9	4.0	2122	19.7	0.2	1257	22.6	0.2
Hungary	4854			4854												
Iceland	363			363			93.4	6.6	0.0	0.0	114	3.5	0.0	86	1.2	0.0
Italy	33 832			33 832			34.1	48.9	16.0	1.1	6029	15.2	0.4	3486	6.6	0.1
Kazakhstan	3184	2974		210	100.0	0.0					640	16.3	0.5	640	16.0	1.3
Latvia	601	350	251	0	58.2	41.8	59.4	39.6	1.0	0.0	161	10.6	0.0	162	7.6	0.0
Lithuania	857	192	68	597	73.8	26.2	12.6	56.6	30.9	0.0	88	26.6	3.8	6	16.7	0.0
Luxembourg	487	483	4	0	99.2	0.8	43.9	56.1	0.0	0.0	137	19.0	0.0	74	8.1	0.0
Malta	197	197	0	0	100.0	0.0					47	2.1				
Moldova	701			701												
Montenegro	454	362	92	0	79.7	20.3	27.1	38.8	33.5	0.7	109	27.5	1.8	20	35.0	0.0
North-Macedonia	1889	1440	449	0			31.3	61.8	6.9	0.0	559	8.8	0.0	86	18.6	0.0
Norway	5085			5085												
Poland	9431	5489	3888	54	58.5	41.5	55.9	43.4	0.7	0.0	2368	11.5	0.2	2288	6.6	0.2
Portugal	4252	3032	1220	0	71.3	28.7	38.0	61.1	0.8	0.0	1080	18.6	0.2	575	14.0	0.2
Romania	1792	630	1162	0	35.2	64.8	42.2	48.9	8.3	0.6	563	14.9	0.4	384	14.6	0.3
Russia	62 891	14 732	35 018	13 141	29.6	70.4	43.4	55.1	1.5	0.0	16 163	16.8	0.4	9973	15.1	0.2
Serbia	176	176	0	0	100.0	0.0	20.5	30.1	49.4	0.0	56	28.6	1.8			
Slovenia	2449	1339	1110	0	54.7	45.3	57.1	42.8	0.1	0.0	637	7.8	0.0	345	7.5	0.0
Spain	27 351	21 884	5467	0	80.0	20.0	37.2	60.3	2.5	0.0	7122	15.7	0.1	6805	13.0	0.2
Sweden	8956	6226	2730	0	69.5	30.5	84.2	15.8	0.0	0.0	2656	3.5	0.0	2301	2.2	0.0
Switzerland	3945	2425	1520	0	61.5	38.5	44.4	50.7	4.9	0.0	1007	17.6	0.2	881	13.4	0.5
The Netherlands	10 715			10 715												
Ukraine	5993	1464	4529	0	24.4	75.6	36.6	54.9	8.4	0.0	2143	16.9	0.0	3428	20.8	0.2
UK	35 999	13 060	22 939	0	36.3	63.7	58.6	38.7	2.7	0.0	11 695	10.5	0.1	6373	10.2	0.2
All*	389 434	167 214	131 975	90 245	55.9	44.1	46.0	49.2	4.5	0.3	91 024	14.2	0.3	59 588	11.2	0.2

* Totals refer only to these countries where data on number of transferred embryos and on multiplicity were reported.

As a result of decreasing numbers of embryos replaced per transfer, the proportion of both twin and triplet deliveries continued to decrease (Fig. 2B). In 2017, twin and triplet rates for fresh IVF and ICSI cycles together were 14.2% (range 2.1–28.6) and 0.3% (range: 0–1.8), respectively. Corresponding results for FER were 11.2% and 0.2%. In the three countries with rates of single embryo replacement above 80% in fresh cycles (93.4% for Iceland, 91.3% for Finland and 84.2% for Sweden), twin rates were as low as 3.5% (for Iceland and Sweden, not available for Finland).

Additional information on pregnancies and deliveries is provided in Supplementary Tables SXIII and SXIV. The reported incidence of pregnancy loss was 16.6% (16.4% in 2016) after IVF+ICSI and 18.3% (18.6% in 2016) after FER. The proportion of recorded lost to follow-up was 7.5% (7.8% in 2016) after IVF+ICSI and 8.1% (7.5% in 2016) after FER.

### Perinatal risks and complications

**Figure 3.**
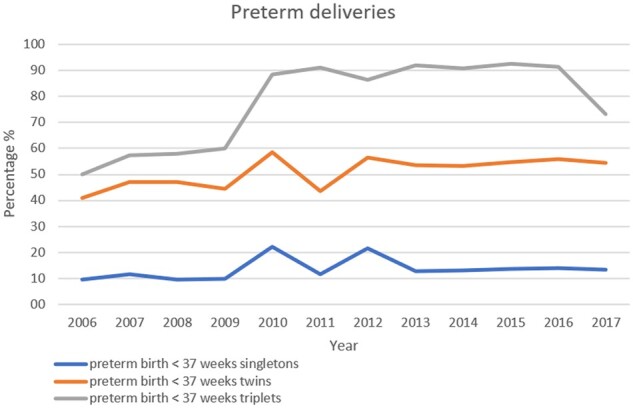
**Proportion of premature deliveries (<37 weeks of gestation in relation to pregnancies ≥37 week of gestation) in singleton, in twin and in triplet pregnancies in Europe, 2006–2017**.

Data on premature deliveries in 2017 were available from 19 countries (18 countries in 2016). Premature DR pooled for fresh IVF and ICSI, FER and ED and according to multiplicity are presented in Supplementary Table SXV. The incidence of extreme preterm birth (20–27 gestational weeks at delivery) was 1.1% in singletons (1.1% in 2016), 3.4% in twins (3.3% in 2016) and 10.7% in triplets (8.4% in 2016). Very premature birth rates (28–32 gestational weeks at delivery) were recorded in 2.4% of singletons (2.2% in 2016), 10.3% of twin pregnancies (10.5% in 2016) and 21.7% in triplet pregnancies (in 2016: 45%). Proportions of premature deliveries before 37 weeks according to multiplicity are shown Fig. 3. Term deliveries (≥37 weeks) were achieved in 86.6% (85.9% in 2016) of singleton pregnancies, 45.2% (44.1% in 2016) of twin pregnancies and 27% (8.8% in 2016) of triplet pregnancies.

Complications related to oocyte retrievals were reported by 32 countries (33 in 2016) and foetal reductions by 24 countries (26 in 2015) (Supplementary Table SXVI). The total reported number of OHSS (Grades 3–5) was 1839 (1928 in 2016) corresponding to a reported incidence of 0.20% (0.21% in 2016). Other complications were less frequent (1484; 1471 cases in 2016) with a total reported incidence of 0.16% (0.2% in 2016), with bleeding being the most reported (0.1%, identical to 2016). In 2017 one maternal death was reported (none in 2016). Foetal reductions were reported in 599 cases (553 in 2016), the majority from UK, Belgium and Spain, as in 2016.

### PGT/PGT-A

Table I includes PGT and PGT for aneuploidies (PGT-A) activities, which were reported from 25 countries (22 in 2016, 23 in 2015). The main contributors were Spain, Russia and Italy. The number of treatment cycles amounted to 37 303 representing 4.3% of initiated IVF+ICSI and FER cycles together (27 069; 3.3% in 2016). More details on PGT/PGT-A activities can be found in the annual reports of the ESHRE PGT Consortium (Coonen *et al.*, 2020). These involved 24 120 fresh cycles and 13183 thawings, resulting in 5109 fresh and 11 205 frozen embryo transfers. In total, 1995 pregnancies (39.0% per transfer) and 1685 deliveries (33.0% per transfer) resulted from fresh cycles. Corresponding figures for FER were 5601 (50.0% per transfer) and 4302 (38.4% per transfer).

### IVM

A total of 378 treatments with IVM were reported from eight countries (654 treatments from eight countries in 2016) (Table I). Most IVM cycles were recorded in Belgium (in Russia for 2016). A total of 184 transfers resulted in 51 pregnancies (27.7% per transfer) and 31 deliveries (16.8% per transfer).

### FOR

A total number of 5210 thawing cycles were reported by 21 countries (4878 from 15 countries in 2016) (Table I) with Italy and Spain being the largest contributors (1391 and 798 cycles, respectively). Amongst 3951 transfers, 1086 resulted in pregnancies (27.5%; 29.5% in 2016) and 831 in deliveries (21%; 21% in 2016).

### IUI

Data on IUI with husband semen (IUI-H) or using donors’ semen (IUI-D) were collected by a total of 1273 institutions (1197 in 2016) in 30 and 25 countries (28 and 23 in 2016), respectively (Table V). Amongst 155 794 IUI-H (162 948 in 2016) and 51 402 IUI-D (50 467 in 2016) reported cycles, the numbers were the highest for IUI-H in Spain, Italy and Belgium, and for IUI-D in Spain, Denmark and Belgium (Supplementary Tables SXVII and SXVIII).

**Table V:** IUI with husband (IUI-H) or donor (IUI-D) semen in 2017.

	IUI-H						IUI-D					
Country	Cycles	Deliveries	Deliveries (%)	Singleton (%)	Twin (%)	Triplet (%)	Cycles	Deliveries	Deliveries (%)	Singleton (%)	Twin (%)	Triplet (%)
Albania	57	4	7.0	50.0	50.0	0.0						
Armemia	1020	249	24.4	95.7	4.3	0.0	207	87	42.0	90.5	9.5	0.0
Austria	2156	194	9.0				386	42	10.9	95.2	2.4	2.4
Belarus	1264	111	8.8	91.8	8.2	0.0	42	4	9.5	100.0	0.0	0.0
Belgium	11 892	780	6.6	95.0	4.9	0.1	8759	902	10.3	96.1	3.8	0.1
Bosnia-Herzegovina	193	14	7.3	92.9	7.1	0.0						
Bulgaria	1161	103	8.9	91.3	8.7	0.0	291	24	8.2	87.5	12.5	0.0
Croatia	1640	146	8.9	100.0	0.0	0.0						
Czech Republic												
Denmark	10 630	1159	10.9	89.0	10.9	0.1	9120	539	5.9	94.1	5.8	0.2
Estonia	86	7	8.1	100.0	0.0	0.0	153	8	5.2	100.0	0.0	0.0
Finland	2433	223	9.2				941	117	12.4			
France	46 395	4888	10.5	90.6	9.1	0.3	2972	588	19.8	90.8	8.9	0.3
Germany												
Greece	3320	153	4.6	98.0	2.0	0.0	336	34	10.1	100.0	0.0	0.0
Hungary												
Iceland	22	3	13.6	100.0	0.0	0.0	176	24	13.6	100.0	0.0	0.0
Italy	18 688	1286	6.9	91.8	7.7	0.5	743	110	14.8	85.5	13.6	0.9
Kazakhstan												
Latvia	120	5	4.2	100.0	0.0	0.0	69	3	4.3	100.0	0.0	0.0
Lithuania	821	32	3.9	100.0	0.0	0.0	4	1	25.0	100.0	0.0	0.0
Luxembourg	322	45	14.0	97.8	2.2	0.0	105	9	8.6	100.0	0.0	0.0
Malta												
Moldova												
Montenegro	201	14	7.0	85.7	7.1	7.1						
North-Macedonia	1216	99	8.1	97.0	3.0	0.0	120	21	17.5	100.0	0.0	0.0
Norway	278	20	7.2	95.0	5.0	0.0	736	106	14.4	97.2	2.8	0.0
Poland	11 418	688	6.0	94.2	5.5	0.3	1995	218	10.9	96.8	3.2	0.0
Portugal	2123	187	8.8	89.8	9.6	0.5	405	72	17.8	84.5	14.1	1.4
Romania	1364	89	6.5	97.4	2.6	0.0	120	15	12.5	93.3	6.7	0.0
Russia	7857	708	9.0	93.9	5.7	0.4	3201	376	11.7	92.6	7.2	0.3
Serbia	434	27	6.2	96.3	3.7	0.0						
Slovenia	591	44	7.4	95.5	4.5	0.0						
Spain	22 199	2122	9.6	89.8	9.8	0.3	12 765	1915	15.0	90.2	9.7	0.1
Sweden							1818	281	15.5	96.1	3.9	0.0
Switzerland												
The Netherlands												
Ukraine	1636	145	8.9	95.9	4.1	0.0	348	41	11.8	92.7	7.3	0.0
UK	4257						5590	816	14.6	95.0	4.8	0.2
All*	155 794	13 545	8.9	91.6	8.1	0.3	51 402	6353	12.4	92.9	6.9	0.2

* Total refers to these countries where data were reported and mean percentage was computed on countries with complete information.

DR could be calculated for 151 537 IUI-H cycles (8.9% as in 2016) and 51 402 for IUI-D cycles (12.4% vs 12.4% in 2016).

Singleton deliveries were the most frequent regardless of the age group with an overall rate of 91.6% for IUI-H and 92.9% for IUI-D (91.0% in IUI-H, 91.9% in IUI-D in 2016). Twin and triplet rates were 8.1% and 0.3%, respectively, for IUI-H, and 6.9% and 0.2% for IUI-D, respectively (in 2016: 8.8% and 0.3%, respectively, for IUI-H and 7.7% and 0.4%, respectively, for IUI-D).

### Sum of fresh and FER (‘cumulative’) DR

Supplementary Table SXIX provides an estimate of a cumulative DR (different from a true cumulative DR, which is based on all transfers resulting from one aspiration). It was calculated as the ratio between the total number of deliveries from fresh embryo transfers and FER performed during a year (numerator) and the number of aspirations during the same year (denominator). The calculation included data from 34 countries (38 countries in 2016) where an overall rate of 30.8% (29.6% in 2016) was recorded. The gain taken from additional FER (over DR from fresh embryo transfers) was 12.3% (10.5% in 2016), with the highest benefits recorded for Ukraine (+29.8%), Finland (+21.1%), Sweden (+20.2%), Armenia (+19.7%) and Latvia (+19.2%), and the lowest for Serbia (0%), Montenegro (+3.4%), North-Macedonia (+3.8%), Bulgaria (+5.8%) and Belarus (+6.2%).

### Cross-border reproductive care

Eight countries reported data on cross-border patients: Albania, Belarus, Denmark, Poland, Portugal, Slovenia, Spain and Switzerland. A total of 16 733 cycles (19 239 in 2016) were reported, 16.9% (22.1% in 2016) of which involved IVF/ICSI with the couple’s own gametes, 50.1% (46.6% in 2016) were EDs and 25.8% (21.8% in 2016) were IVF or ICSI with semen donation. Additionally 7298 IUI with sperm donation (7062 in 2016) were registered. Information regarding the countries of origin was very incomplete and not reliable enough to obtain any conclusive information. The main reasons reported by patients were to have access to a technique not legally available in their home country (41.9%; 39.1% in 2016) or to seek a higher quality treatment (25.7%; 23.6% in 2016). In 13 551 cycles (mainly from Spain), there was another, not specified, reason to travel abroad.

### FP

For the second year data on FP were reported. Fourteen countries (11 in 2016) provided data on a total number of 18 888 interventions (13 689 in 2016) (Supplementary Table SXX) in pre- and postpubertal patients, both for medical and non-medical reasons. The majority of interventions consisted of the cryopreservation of ejaculated sperm (n = 11 112 from 13 countries; 7877 from 11 countries in 2016) and the cryopreservation of oocytes (n = 6588 from 13 countries; n = 4907 from eight countries in 2016). Ovarian tissue cryopreservation was reported by three (2 in 2016) and 10 (7 in 2016) countries, respectively, for pre- and postpubertal patients, with the use of postpubertal tissue through transplantation reported in three countries (France, Italy and Spain). Testicular tissue cryopreservation in postpubertal patients and prepubertal boys was reported from eight (six in 2016) countries and from four countries (Austria, Belgium, France and Poland), respectively.

## Discussion

This is the 21st annual report on ART, IUI and, for the second time, FP activity data collected by EIM from European compulsory or voluntary registries. From 1997 to 2017, the EIM Consortium of ESHRE has registered over 10 million treatments cycles (10 713 407) that have led to the birth of over 2 million infants.

This report presents the analysis of data collected in 2017 from 39 European countries (40 in 2016). The number of European countries participating has remained quite stable over the last years with only a few non- participating countries (5 of 44 EIM members, 7 non-EIM members including Azerbaijan, Kosovo and 5 countries that do not provide ART services). The main reasons for not being able to send data most likely appear to be either economic at centre and/or country level, regulatory or political (Calhaz-Jorge *et al.*, 2020). Although the participation of some countries has fluctuated over the years, reported ART treatment numbers are still on the rise (+2.4% as compared to 2016) as are the number of infants born from ART (+1.2% compared to 2016).

Considering the importance of data collection to improve clinical care, including vigilance on medically assisted reproduction (MAR treatments) (De Geyter *et al.*, 2016; Kissin, 2019) , EIM and the EU affairs committee of ESHRE are striving to increase awareness of country competent authorities and EU DG SANTE, respectively, aiming at reaching the highest level of completeness and harmonization of European data on reproductive care. Amongst other actions, country representatives and competent authorities have been invited to ESHRE activities related to data collection (https://www.eshre.eu/ESHRE2021/Programme/Precongress-Courses/Course-14-EIM). Despite known challenges in data collection and after exclusion of countries where ART is not available, the participation rate at the country level is as high as 86.3% of European countries (88.7% of EIM members) while at the level of IVF clinics the proportion of those reporting data is at 90.1% (vs 91.8% in 2016; Wyns et *al.*, 2020).

As previously, levels of completeness of the data are variable amongst countries but 21 countries were able to send data from all IVF clinics (20 in 2016, 18 in 2015 and 14 in 2014). Besides reaching a higher number of countries able to provide complete data sets, obtaining cycle-by-cycle data (15 countries in 2017) should be the next priority to facilitate data interpretation and reliability.

Further progress towards higher quality of the data is expected through harmonization of reported data by registries. In this regard, core data sets on outcome parameters with definitions of collected items have been established (https://www.eshre.eu/Data-collection-and-research/Consortia/EIM). Collecting higher quality data is also in line with future requirements and expectations at the EU level. Meanwhile, owing to the variety of collection systems, the absence or limited presence of data validation methods and quality control, and differences in definitions and country or centre-specific practices (e.g. freeze all cycles, embryo transfer policy, PGT-A), interpretation of the data should remain cautious.

Being aware of the current EU objective of increased transparency on MAR care for all stakeholders, including the patient, data on availability and cross-border care are of utmost importance. Over the years, EIM has constantly recorded a high variability in access to treatment between countries, ranging from 723 to 3286 per million inhabitants and 3893 to 15 783 per million females of reproductive age in 2017.

So far, data generated by EIM on numbers of treatment cycles per million inhabitants and per woman of reproductive age by country are unique in Europe and very relevant to assess equity in access to infertility care. Currently and as in 2016, 60% of countries with complete data sets reach the historical estimated threshold of 1500 fresh ART cycles needed for infertility care per million inhabitants (The ESHRE Capri Workshop Group, 2001). However, owing to technological evolutions in ART with increasing success rates and higher numbers of FER treatment cycles over time (Fig. 1) (representing 48.9% of ART cycles when fresh IVF+ICSI cycles are used as the denominator vs 44.1% in 2016), such estimates may have become obsolete and deserve to be reassessed. In this regard, and to eliminate age differences amongst countries, estimates should preferably use the number of women of reproductive age as denominator. Moreover, as data on cross-border patients were available for only eight countries (10 in 2016) such a low reporting represents another serious limitation when it comes to estimating access to care.

Regarding treatment modalities, while ICSI remains the most applied with a trend to stabilization of its use during recent years (Table I andFig. 1), FER is the second most used technique. A progressive increase in the proportion of FER relative to fresh IVF and ICSI cycles has also been recorded over the years (37.8% in 2014, 40.3% in 2015, 44% in 2016 and 48.9% in 2017). The high variability in the proportion of FER cycles amongst countries that both report FER cycles and provide complete data sets (ranging from 27.5% to 96.3%), reflects variable practices in terms of stimulation protocols, embryo transfer policy and embryo cryopreservation. Amongst others, freeze-all cycles, now registered since 2014 (Supplementary Table SV) and increasingly reported, show proportions per aspiration ranging between 2.3% and 49.1% for embryos. Such differences should not be neglected when analyzing data. For instance, lower overall pregnancy and DR for fresh IVF and ICSI cycles (per aspiration) compared to FER cycles (per thawing) (Table III) need to be cautiously interpreted taking into account variability in practices, including freeze-all cycles. Other evolutions, such as increased frequency of vitrified-warmed blastocyst transfers, could also explain better outcomes after FET.

Cumulative DR per cycle or per aspiration are therefore better outcome indicators to assess treatment effectiveness (De Neubourg *et al.*, 2016). So far, the EIM consortium has gathered aggregated data that preclude the calculation of true cumulative delivery and livebirth rates. Hence, a proxy-indicator for true cumulative rates, based on the addition of outcomes of fresh and FER during the same calendar year, has been considered. Taking into account data from 34 countries, cumulative DR reached 30.8% (29.6% in 2016) during the 1-year period.

Important trends have been noted over time in EIM data sets (Ferraretti *et al.*, 2017; De Geyter *et al.*, 2020). Amongst these, a reduction in the number of replaced embryos per transfer (Fig. 2A) and of multiple births (Fig. 2B) were highlighted. As a result of promoting a higher awareness amongst professionals, the proportion of transfers of only one embryo (whether elective or not) continues to rise (46% vs 41.5% in 2016), and the number of transfers of three or more embryos continues to decrease. Paralleling this trend, multiple birth rates decrease, with the lowest rates observed (3.5%) when the proportion of single embryo transfers is above 80%. As recorded twin and triplet rates were slightly lower for FER, as in 2016, the increase in FER could also have influenced multiple birth rates. The impact of foetal reductions remains, however, unknown: 61% of the 39 countries in 2017 reported data on foetal reductions, but only a limited number of the countries (17) actually performed them.

In the future, it is highly expected that efforts will lead to the ultimate goal of the birth of one healthy child (Land and Evers, 2003) per embryo transfer and to a reduction of prematurity associated with multiple births. Looking at the evolution of the proportions of deliveries before 37 weeks according to multiplicity (Fig. 3), it is rather clear that besides efforts to reduce triplets, the focus needs to be on reducing twins where preterm births are still as high as 45.2%, and extreme and very preterm births increased by 3- and 4-folds compared to singletons, respectively.

Aiming at elective single embryo transfer but also at a reduced time to pregnancy, the field has focused on prolonging embryo culture up to the blastocyst stage. For the second time, in 2017 the proportion of transfers at the blastocyst stage was collected and showed a slight increase since 2016 (44.1% vs 41.9% for fresh transfers and 64.1% vs 62.2% for FER). While the benefit of blastocyst stage transfers on ART outcomes is still a matter of debate (Glujovsky *et al.*, 2016; Practice Committee of ARSM, 2018), EIM data showed PR for blastocyst transfers to be higher (41.7% vs 29.4% for cleavage stage embryos for IVF and ICSI and 38.5% vs 27.2% for cleavage stage embryos for FER). Unfortunately, data on deliveries by stage of embryo transfer were not available.

With regard to the safety aspects of ART treatments, besides multiplicity and prematurity, the reporting remains most likely poor with the highest rate of complications for ovarian hyperstimulation syndrome (0.2% similar to 2016) and a total incidence of other complications at 0.16%. It is, however, noticeable that there was one maternal death after ART reported in 2017 (Supplementary Table SXVI).

The future of MAR registries should focus on levelling up the quality of collected data towards complete and harmonized data allowing comparisons of practices and identification of the safest and most efficient care. The next priorities should include the health of the still increasing numbers of children born from ART considering also that evolution in the reproductive field will most likely be marked by increasing needs for ART, owing to FP interventions linked to postponement of motherhood or gonadotoxic therapies and further developments in PGT.

## Data availability

All data are incorporated into the article and its online supplementary material.

## Authors’ roles

C.W. drafted the manuscript and was responsible for final editing of the manuscript. C.D.G., C.C.-J., M.S.K., T.M., J.S., C.B., A.T.-S., I.A.R. and S.V. edited the manuscript. V.G. was responsible for the data collection and edited the manuscript. V.G. was responsible for raw data curation, contributed to the tables, contributed to the figures and edited the manuscript. All authors revised and approved the final manuscript.

## Funding

The study has received no external funding and all costs are covered by ESHRE.

## Conflict of interest

There are no competing interests.

## Supplementary Material

Supplementary_Table_S1_final

Supplementary_Table_S2_final

Supplementary_Table_S3_final

Supplementary_Table_S4_final

Supplementary_Table_S5_final

Supplementary_Table_S6_final

Supplementary_Table_S7_final

Supplementary_Table_S8_final

Supplementary_Table_S9_final

Supplementary_Table_S10_final

Supplementary_Table_S11_final

Supplementary_Table_S12_final

Supplementary_Table_S13_final

Supplementary_Table_S14_final

Supplementary_Table_S15_final

Supplementary_Table_S16_final

Supplementary_Table_S17_final

Supplementary_Table_S18_final

Supplementary_Table_S19_final

Supplementary_Table_S20_final

## Appendix

Contact persons who are collaborators and represent the data collection programmes in participating European countries, 2017.

**Albania**

Prof. Orion Gliozheni, University Hospital for Obst & Gynecology, Department of Obstetrics & Gynecology, Bul.B.Curri, Tirana, Albania. Tel: +355 4 222 36 32; Fax: +355 4 2257 688; Mobile: +355 68 20 29 313. E-mail: glorion@abcom.al

**Armenia**

Mr Eduard Hambartsoumian, Fertility Center, IVF Unit, 4 Tigvan Nets, 375010 Yerevan, Armenia; Tel: +374 10 544368; E-mail Hambartsoumian@hotmail.com

**Austria**

Prof. Dr Heinz Strohmer, Dr Obruca & Dr Strohmer Partnerschaft Goldenes Kreuz-Kinderwunschzentrum, Lazarettgasse 16-18, 1090 Wien, Austria. Tel: +43 401 111 400; Fax: +43 401 111 401. E-mail: heinz.strohmer@kinderwunschzentrum.at

**Belarus**

Dr Elena Petrovskaya (Alena Piatrouskaya), ART Centre ‘Embryo’, Filimonova 53, 220053 Minsk, Belarus. Tel: +375 293 830 570; E-mail: elenaembryoby@gmail.com

Dr Oleg Tishkevich, Centre For Assisted Reproduction “Embryo” Belivpul, Filimonova Str. 53, 220114 Minsk, Belarus. Tel: +375 296 222 722; Fax: +375 172 376 404; Mobile: +375 296 222 722; E-mail: tishol@tut.by

**Belgium**

Dr Kris Bogaerts, I-Biostat, Kapucijnenvoer 35 bus 7001, 3000 Leuven, Belgium. Tel: +32 (0) 16 33 68 90; Fax: +32 (0) 16 33 70 15. E-mail: Kris.Bogaerts@med.kuleuven.be

Prof. Christine Wyns, Gynaecology-Andrology, Cliniques Universitaires Saint Luc, Service FIV- Andrology, Université Catholique de Louvain; Av. Hippocrate, 10, 1200 Brussels, Belgium. Tel: +32 27646576; Fax +32 27649050; Mobile +32 477943374; E-mail: christine.wyns@uclouvain.be

**Bosnia**

Prof. Dr Devleta Balic, Zavod za humanu reprodukciju ‘Dr Balic’, Kojsino 25, 75000 Tuzla, Bosnia—Herzegovina. Tel: +387 35 260 650; Mobile: +387 611 402 22; E-mail drbalic@bih.net.ba

Prof. Dr Sanja Sibincic, Health Center Medico-S, Jevrejska 58/A, 78000 Banja Luka, Bosnia—Herzegovina. Tel: +387 512 321 00; Mobile: +387 655 159 42; E-mail sanjasibincic@gmail.com

**Bulgaria**

Irena Antonova, ESHRE certified clinical embryologist (2011), Ob/Gyn Hospital Dr Shechterev, 25-31, Hristo Blagoev Strasse, 1330 Sofia, Bulgaria. Tel: +359 887 127 651; E-mail: irendreaming@gmail.com

**Croatia**

Prof. Dr Hrvoje Vrcic, Zagreb University Medical School, Obstetrics and Gynecology, Petrova 13, 10000 Zagreb, Croatia. Tel: +385 146 046 46; Fax: +385 146 335 12; E-mail: Hrvoje.vrcic@hilarus.hr

Dr Dejan Ljiljak, Clinical Hospital Center ‘Sestre milosrd’, Department for Biology of Human Reproduction, Ob/Gyn Clinic, Vinogradska c. 29, 10000 Zagreb, Croatia. Tel: +385 378 75 97; Fax: +385 137 682 72; Mobile: +385 3787 125; E-mail: dejan.ljiljak@kbcsm.hr

**Czech Republic**

Dr Karel Rezabek, Medical Faculty, University Hospital, CAR-Assisted Reproduction Center, Gyn/Ob Department, Apolinarska 18, 12000 Prague, Czech Republic. Tel: +420 224 967 479; Fax: +420 224 922 545; Mobile: +420 724 685 276; E-mail: rezabek.ivf@seznam.cz

Mgr. Jitka Markova, Institute of Health Information and Statistics of the Czech Republic, Palackeho namesti 4, 12801 Prague, Czech Republic. Tel: +420 224 972 832; Mobile: +420 721 827 532; E-mail: jitka.markova@uzis.cz

**Denmark**

Dr Josephine Lemmen, Vitanova, Fertility clinic, Vester Voldgade 106, 1552 Copenhagen, Denmark. Tel: +45 333 371 01; E-mail: jglemmen@gmail.com

**Estonia**

Dr Deniss Sõritsa, Tartu University Hospital and Elitre Clinic, Tartu, Estonia. Tel: +372 740 9930; Fax: +372 740 9931; E-mail: soritsa@hotmail.com

**Finland**

Prof. Mika Gissler, THL National Institute for Health and Welfare, P.O. Box 30, 00271 Helsinki, Finland. Tel: +385 29 524 7279; E-mail: mika.gissler@thl.fi

Dr Sari Pelkonen, Oulu University Hospital, Department of Obstetrics and Gynaecology, P.O. Box 23, 90029 Oys, Finland. Tel:+358 8 3153040, E-mail sari.pelkonen@fimnet.fi

**France**

Dr Bilal Majed, Agence de la biomedecine, 1 av stade de France, 93 Saint Denis, France; E-mail: bilal.majed@biomedecine.fr

Prof. Jacques de Mouzon, 15-29 rue Guilleminot, 75014 Paris, France. Tel: +33 143 224 679; Mobile: +33 662 062 274; E-mail: jacques.de.mouzon@gmail.com

**Germany**

Dr Andreas Tandler—Schneider; Fertility Center Berlin; Spandauer damm 130; 14050 Berlin; Germany. Tel: +49 30 233 20 81 10; Fax: +49 30 233 20 81 19; E-mail: tandler-schneider@fertilitycenter-berlin.de

**Greece**

Prof. Nikos Vrachnis, National Authority of Medically Assisted Reproduction, Ploutarxou 3, P.O. 10675 Athens. Tel: +30 6974441144; E-mail: nvrachnis@hotmail.com

**Hungary**

Prof. Janos Urbancsek, Semmelweis University, 1st Department of Ob/Gyn, Baross utca 27, 1088 Budapest, Hungary. Tel: +36 1 266 01 15; Fax: +36 1 266 01 15; E-mail: urbjan@noi1.sote.hu

Prof. G. Kosztolanyi, University of Pecs, Department of Medical Genetics and Child Development, Jozsef A.u; 7., 7623 Pecs, Hungary. Tel: +36 7 2535977; Fax: +36 7 2535972; E-mail: gyorgy.kosztolanyi@aok.pte.hu

**Iceland**

Mr Hilmar Bjorgvinsson, IVF Klinikin Reykjavik, Alfheimum 74, 104 Reykjavik, Iceland. Tel: +354 430 4000; Fax: +354 430 4040; E-mail: Hilmar.bjorgvinsson@ivfklinikin.is

**Italy**

Dr Giulia Scaravelli, Istituto Superiore di Sanità, Registro Nazionale della Procreazione Medicalmente Assistita, CNESPS, Viale Regina Elena, 299, 00161 Roma, Italy. Tel: +3906 499 04 050; Fax: +39064 99 04 324; E-mail: giulia.scaravelli@iss.it

Dr Roberto de Luca, Istituto Superiore di Sanità, Registro Nazionale della Procreazione Medicalmente Assistita, CNESPS, Viale Regina Elena, 299, 00161 Roma, Italy. Tel.: +3906 499 04 320; E-mail: roberto.deluca@iss.it

**Kazakhstan**

Prof. Dr Vyacheslav Lokshin, International Clinical Center for Reproductology ‘Persona’, Utepova street 32a, 00506 Almaty, Kazakhstan. Tel: +7 727 382 7777; Mobile: +7 701 755 8209; E-mail: v_lokshin@persona-ivf.kz

Dr Sholpan Karibayeva, International Clinical Center for Reproductology ‘Persona’, Utepova Street 32a, 00506 Almaty, Kazakhstan. Tel: +7 727 382 7777; E-mail: sh.karibaeva@gmail.com

**Latvia**

Dr Valeria Magomedova, Jusu Arsti Private Clinic, Apuzes 14, 1046 Riga, Latvia. Tel: +371 678 700 29; Fax: +371 678 704 29; E-mail: godunova@inbox.lv

**Lithuania**

Raminta Bausyte, Vilnius University Hospital Santaros Clinics, Santaros Fertility Center, Simono Staneviciaus 64-69, 07113 Vilnius, Lithuania. Tel: +370 620 86826; E-mail: raminta.bausyte@gmail.com

Ieva Masliukaite, Academic Medical Center, Cener for Reproductive Medicine, Ijburglaan 1086ZJ Amsterdam, The Netherlands. Tel: +31 653 688 815; E-mail: i.masliukaite@amc.uva.nl

**Luxembourg**

Dr Caroline Schilling, Centre Hospitalier de Luxembourg, Centre de Stérilité et de Médecine de Reproduction, Rue Fiederspiel 2, 1512 Luxembourg, Luxembourg. Tel: +352 44 11 32 30; Mobile: +352 66 13 13 912; E-mail: schilling.caroline@chl.lu

**Malta**

Dr Jean Calleja-Agius, University of Malta, 12, Mon Nid, Gianni Faure Street, TXN2421 Tarxien, Malta. Tel: +356 216 930 41; Mobile: +356 995 536 53; E-mail: jean.calleja-agius@um.edu.mt

**Moldova**

Prof. Dr Veaceslav Moshin, Medical Director at Repromed Moldova, Center of Mother @ Child Protection, State Medical and Pharmaceutical University ‘N.Testemitanu’, Bd. Cuza Voda 29/1, Chisinau, Republic of Moldova. Tel: +37322 263855; Mobile: +37369724433; E-mail: mosin@repromed.md; veaceslavmoshin@yahoo.com

**Montenegro**

Dr Tatjana Motrenko Simic, Human Reproduction Center Budva, Prvomajska 4, 85310 Budva, Montenegro. Tel: +382 33402432; Mobile: +382 69 052 331; E-mail: motrenko@t-com.me

Dragana Vukicevic, Hospital ‘Danilo I’, Humana reprodukcija, Vuka Micunovica bb, 86000 Cetinje, Montenegro. Tel: +382 675 513 71; E-mail: vukicevic.dragana@yahoo.com

**The Netherlands**

Dr Jesper M.J. Smeenk, Department of Obstetrics and Gynaecology, St. Elisabeth Hospital Tilburg, Hilv, The Netherlands. Tel: +31 13 539 31 08; Mobile +31 622 753 853; E-mail: j.smeenk@elisabeth.nl

**North Macedonia**

Mr Zoranco Petanovski, Hospital ReMedika, Nas. Zelezara, 1000 Skopje, Macedonia. Tel: +389 224 475 45, Fax: +389 226 031 00; E-mail: zpetanovski@yahoo.com

**Norway**

Dr Liv Bente Romundstad, Spiren Fertility Clinic, Nardoskrenten 11, 7032 Trondheim, Norway. Tel: +47 73523000; Mobile +47 90550207; E-mail libero@klinikkspiren.no

**Poland**

Dr Anna Janicka, VitroLive, Wojska Polskiego 103, 70-483 Szczecin, Poland. Tel: +48 91 88 69 260; E-mail anna.janicka@vitrolive.pl

**Portugal**

Prof. Dr Carlos Calhaz—Jorge, CNPMA, assembleia da Republica, Palacio de Sao Bento, 1249-068 Lisboa, Portugal. Tel: +351 21 391 93 03; Fax: +351 21 391 75 02; E-mail: calhazjorgec@gmail.com

Ms Joana Maria Mesquita Guimaraes. Hospital Geral Santo Antonio, Largo Professor Abel Salazar, 4050-011 Porto, Portugal. Tel: +351 96 616 02 37; E-mail: joanamesquitaguimaraes@gmail.com

Ms Ana Rita Laranjeira, CNPMA, Assembleia da Republica, Palaio de Sao Bento 1249-068 Lisboa, Portugal. Tel +351 21 391 93 03; Fax +351 21 391 75 02; E-mail cnpma.correio@ar.parlamento.pt

**Romania**

Mrs Ioana Rugescu, Gen Secretary of AER Embryologist Association and Representative for Human Reproduction Romanian Society. Tel: +40744500267; E-mail: irugescu@rdsmail.ro

Dr Bogdan Doroftei, University of Medicine and Pharmacy Iasi; Teaching Hospital Obgyn ‘Cuza Voda’; Cuza Voda Str. 34, 700038 Iasi, Romania. Tel: +40 232 213 000/int. 176; Mobile: +40 744 515 297; E-mail bogdandoroftei@gmail.com; bogdan.doroftei@umfiasi.ro

**Russia**

Dr Vladislav Korsak, International Center for Reproductive Medicine, General Director, Liniya 11, Building 18B, Vasilievsky Island, 199034 St-Petersburg, Russia C.I.S. Tel: +7 812 328 2251; Fax: +7 812 327 19 50; Mobile: +7 921 9651977; E-mail: korsak@mcrm.ru

**Serbia**

Prof. Snezana Vidakovic, Institute for Obstetrics and Gynecology, Clinical Centre ’GAK’, Visegradska 26, 11000 Belgrade, Serbia. Tel: +381 63 24 23 80; E-mail: drvidakovicsnezana@gmail.com

**Slovenia**

Prof. Irma Virant-Klun, University Medical Centre Ljubljana, Department of Obstetrics and Gynecology, Slajmerjeva 3, 1000 Ljubljana, Slovenia. Tel: +386 1 522 60 13; Fax: +386 1 431 43 55; Mobile: +38631625774; E-mail: irma.virant@kclj.si

**Spain**

Mrs Irene Cuevas Saiz, Hospital General de Alicante, Department of Infertility, Av Pintor Baeza, 12, 03010 Valencia, Spain. Tel: +34 961972000; Fax: +34 91 799 4407; Mobile: +0034677245650; E-mail cuevas_ire@gva.es

Dr Fernando Prados Mondéjar, Hospital de Madrid-Montepríncipe, HM Fertility Center Monteprincipe, C/Montepríncipe 25, 28660 Boadilla del Monte, Spain. Tel: +34 917 089 931; Mobile +34 646 737 237; E-mail fernandojprados@gmail.com

**Sweden**

Prof. Christina Bergh, Department of Obstetrics and Gynaecology, Sahlgrenska University Hospital, Bla Straket 6, 413 45 Göteborg, Sweden. Tel: +4631 3421000, +46736 889325; Fax: +4631 418717; Mobile: +46 736 889325; E-mail Christina.bergh@vgregion.se

**Switzerland**

Ms Maya Weder, Administration FIVNAT, Postfach 754, 3076 Worb, Switzerland. Tel: +41 (0)31 819 76 02; E-mail: fivnat@fivnat-registry.ch

Dr med. Marco Buttarelli, Centro Cantonale di Fertilità, Ospedale Regionale di Locarno ‘La Carità’, Via all’Ospedale 1, 6600 Locarno, Switzerland. Tel: +41 91 811 45 38; E-mail: fivnat@fivnat-registry.ch

Dr Marie-Pierre Primi, Laboratoire Andrologie et Biologie de la Reproduction (LABR), Unité de Médecine de la Reproduction, Maternité 07, CHUV, 1011 Lausanne, Switzerland. Tel: +41 21 314 34 26; E-mail: fivnat@fivnat-registry.ch

**Turkey**

Dr Basak Balaban, Amerikan Hastanesi—American Hospital, Assisted Reproduction Unit, Güzelbahçe Sok N° 20 Nisantesi, 34365 Istanbul, Turkey. Tel: +90 212 444 3 777; Fax: +90 212 311 2190; Mobile: +90 532 253 27 92; E-mail basakb@amerikanhastanesi.org

Prof. Dr Timur Gürgan, Gurgan Clinic, Infertility, Cankaya caddesi, 20/3, 06680 Cankaya-Ankara, Turkey. Tel: +90 312 4427404; Fax: +90 312 4427407; Mobile: +90 532 231 7706; E-mail: tgurgan@gurganclinic.com.tr

**UK**

Mr Richard Baranowski, Deputy Information Manager, Human Fertilization and Embryology Authority (HFEA), Finsbury Tower, 103-105 Bunhill Row, London EC1 Y 8HF, UK. Tel: +44 (0) 20 7539 3329; Fax: +44 (0) 20 7377 1871; E-mail: Richard.baranowski@hfea.gov.uk

**Ukraine**

Prof. Dr Mykola Gryshchenko, IVF Clinic Implant Ltd, Academician V.I. Gryshchenko Clinic for Reproductive Medicine, 25 Karl Marx Str., 61000 Kharkiv, Ukraine. Tel: +380 57 124522; Fax: +380 57 705070703; Mobile: +380 57 705070703;

E-mail: nggryshchenko@gmail.com
